# Nitrogen Can Alleviate the Inhibition of Photosynthesis Caused by High Temperature Stress under Both Steady-State and Flecked Irradiance

**DOI:** 10.3389/fpls.2017.00945

**Published:** 2017-06-06

**Authors:** Guanjun Huang, Qiangqiang Zhang, Xinghai Wei, Shaobing Peng, Yong Li

**Affiliations:** Ministry of Agriculture Key Laboratory of Crop Ecophysiology and Farming System in the Middle Reaches of the Yangtze River, College of Plant Science and Technology, Huazhong Agricultural UniversityWuhan, China

**Keywords:** dynamic photosynthesis, high temperature, leaf hydraulic conductance, nitrogen, steady-state photosynthesis

## Abstract

Nitrogen is one of the most important elements for plants and is closely related to photosynthesis. High temperature stress significantly inhibits photosynthesis under both steady-state and flecked irradiance. However, it is not known whether nitrogen can affect the decrease in photosynthesis caused by high temperature, especially under flecked irradiance. In the present study, a pot experiment was conducted under two nitrogen (N) supplies with rice plants, and the steady-state and dynamic photosynthesis rates were measured under 28 and 40°C. High temperature significantly increased leaf hydraulic conductance (*K*_leaf_) under high N supply (HN) but not under low N supply (LN). The increased *K*_leaf_ maintained a constant leaf water potential (Ψ_leaf_) and steady-state stomatal conductance (*g*_s,sat_) under HN, while the Ψ_leaf_ and *g*_s,sat_ significantly decreased under high temperature in LN conditions. This resulted in a more severe decrease in steady-state photosynthesis (*A*_sat_) under high temperature in the LN conditions. After shifting from low to high light, high temperature significantly delayed the recovery of photosynthesis, which resulted in more carbon loss under flecked irradiance. These effects were obtained under HN to a lesser extent than under LN supply. Therefore, it is concluded that nitrogen can alleviate the inhibition of photosynthesis caused by high temperature stress under both steady-state and flecked irradiance.

## Introduction

Global warming is threatening crop yields. Lobell and Asner (2003) estimated that each 1°C increase in growing season temperature can result in up to 17% decrease in corn and soybean yield. Experimental data in rice production showed that rice yield decreases by 10% for each 1°C increase in the minimum temperature of the growing-season (Peng et al., 2004). Photosynthesis is important for crop growth and production, and it is very sensitive to temperature. Photosynthesis usually peaks at ~30°C in rice plants, with a significant decrease in CO_2_ assimilation for additional increases in temperature (Yamori et al., 2011).

The intrinsic mechanisms for reduced photosynthesis under high temperature stress have been intensively studied. First, ribulose-bisphosphate carboxylase/oxygenase (Rubisco) is a bifunctional enzyme, and Rubisco activity can be substantially restrained under supra-optimal temperature (Crafts-Brandner and Salvucci, 2000). Moreover, the ratio of the oxygenation to the carboxylation rates of ribulose-bisphosphate (RuBP) increases with increasing temperature, which results in a higher CO_2_ loss via photorespiration (Walker et al., 2013; Gandin et al., 2014). Second, the electron transport rate is substantially inhibited under extreme high temperature stress, i.e., at 40°C (Wise et al., 2004; Sage and Kubien, 2007); Third, the diffusion capacity is very sensitive to temperature, but the responses are species dependent. Stomatal conductance (*g*_s_) and mesophyll conductance (*g*_m_) can increase, decrease, and remain constant with increasing temperature (Yamori et al., 2006; Sage and Kubien, 2007; Scafaro et al., 2011; Evans and von Caemmerer, 2013).

Nitrogen (N) is one of the most important elements and can regulate leaf photosynthesis via several strategies. Firstly, because of the large investments of leaf N to Rubisco and to the electron transport proteins and primary enzymes of the Calvin cycle, leaf photosynthesis is usually positively related to leaf N content. Secondly, *g*_s_ and *g*_m_ can be substantially increased under high N supply (Warren, 2004; Li et al., 2009, 2013; Xiong et al., 2015; Sun et al., 2016). The increment of *g*_m_ under high N supply (HN) is mostly due to increased chloroplastic surface facing the intercellular air spaces (*S*_c_) and decreased cell wall thickness (Li et al., 2009, 2013; Xiong et al., 2015). Moreover, a decreased sink capacity for photosynthate in N-limited plants can lead to carbohydrate accumulation in leaves, which will feedback inhibit photosynthesis (Logan et al., 1999). However, whether and how N supply affects the response of photosynthesis to high temperature stress is not fully understood.

Photosynthesis is mostly studied under saturating and constant light conditions; however, light environments in crop canopies are highly dynamic. Leaves within crop canopies experience a light environment highly variable in magnitude and time throughout the course of a day due to changes in the incoming solar irradiance, wind, cloud cover, and self-shading of the upper leaves (Timm et al., 2002; Lawson et al., 2012). Pearcy et al. (1990) found that sunflecks contribute 20–93% of the total received daily photosynthetic photon flux density (PPFD). Involving many subprocesses, photosynthesis cannot respond linearly to changing irradiance. When the irradiance is shifting from low to high levels, photosynthesis does not immediately increase to the maximum rate, and there is a time lag needed for activation of enzymes and stomatal opening (Lawson et al., 2012; Pearcy and Way, 2012). The incoordination between photosynthesis and fluctuating irradiance significantly decreases the light use efficiency of the dynamic irradiance (Leakey et al., 2002).

The effects of abiotic stresses on dynamic photosynthesis are less well-known. Only a handful of studies have investigated the influences of elevated CO_2_ concentration, water stress, air humidity, and nitrogen supply on dynamic photosynthesis (Leakey et al., 2002; Cui et al., 2009; Sun et al., 2016, 2017). More studies in this field are needed to understand photosynthesis in natural conditions. Leaf temperature can transiently increase when receiving light flecks, relatively brief but high-intensity patches of light, and high temperature can restrain the photosynthetic carbon gain during light flecks (Leakey et al., 2005). To our best knowledge, there is only one study that has investigated the effect of high temperature on dynamic photosynthesis (Leakey et al., 2003). It is demonstrated that high temperature inhibition of photosynthesis is more severe under sunflecks than uniform irradiance in a tropical rain forest tree seedling, *Shorea leprosula*. However, whether and how N supply affects the response of dynamic photosynthesis to high temperature stress are not known.

In the present study, rice plants were grown in pots under low and high N supplies. Both steady-state and dynamic photosynthesis were studied under optimal (28°C) and high (40°C) temperature conditions. The objectives were to study (1) whether nitrogen supply can affect the response of photosynthesis to high temperature stress and (2), if so, what are the intrinsic mechanisms, (3) whether the N supply can affect the response of dynamic photosynthesis to high temperature stress. The findings are important for understanding the interaction between N and temperature on photosynthesis.

## Materials and methods

### Plant materials and N treatments

After germination on moist filter paper on 27 Aug. 2016, seeds of the hybrid rice cultivar *Oryza sativa* L. ssp. indica *cv*. “Shanyou 63” were transferred to nursery plates. When the seedlings had developed an average of 2.5 leaves, they were transplanted to 11.0-L pots with a density of three hills per pot and two seedlings per hill. Each pot was filled with 10.0 kg soil. Phosphorus (P) and potassium (K) were each applied at rates of 1.54 and 1.87 g pot^−1^, respectively. N was applied at a rate of 1.60 g N pot^−1^ under the HN treatment, while no N was applied under the LN treatment. Fertilizers were applied by mixing them into the soil. Plants were watered daily, and a minimum layer of 2 cm of water was maintained to avoid drought stress. Pests were intensively controlled using chemical pesticides. The soil used in this study had the following properties: pH 7.1, 6.7 g kg^−1^ of organic matter, 6.27 mg kg^−1^ of Olsen-P, 129 mg kg^−1^ of exchangeable K, and 0.63‰ total N. The experiment was conducted outdoors at Huazhong Agricultural University (114.37°E, 30.48°N) in Wuhan city, Hubei province, China. The measurements were conducted at the tillering stage, on 3 Oct. 2016.

### Gas exchange measurements

#### Steady-state gas exchange measurements

One day before the gas exchange measurement, the seedlings were moved to a controlled growth chamber (PPFD 1,000 μmol m^−2^ s^−1^ at the leaf level; temperature 28°C; relative humidity 60%; CO_2_ concentration 400 μmol mol^−1^) to avoid environmental fluctuation and midday depression of photosynthesis. Gas exchange measurements were conducted from 9:00 to 15:00 on the newest fully expanded leaves using a portable photosynthesis system (LI-6400XT; LI-COR Inc., Lincoln, NE, USA) with a 6400-40 leaf chamber. Leaves were placed in the leaf chamber for at least 15 min at a PPFD of 1,500 μmol m^−2^ s^−1^ and a CO_2_ concentration in the reference chamber of 400 μmol mol^−1^ with a CO_2_ mixture. Leaf temperatures were controlled at either 28 or 40°C using the gas exchange instrument.

After equilibration to a steady state, the steady-state fluorescence (*F*_s_) was measured and a 0.8-s saturating pulse of light (~8,000 μmol m^−2^ s^−1^) was applied to measure the maximum fluorescence ($F_{\text{m}}^{\prime }$). The gas exchange data were also recorded simultaneously, and they were used to define the steady-state photosynthesis (*A*_sat_), stomatal conductance (*g*_s,sat_) and intercellular CO_2_ concentration (C_i,sat_). The photosynthetic efficiency of photosystem II (Φ_*PSII*_) was calculated as follows:

(1) $$\begin{array}{l} \Phi_{\text{PSII}}=1-\frac{F_{s}}{{F_{m}}^{\prime }} \end{array}$$

The electron transport rate of PSII (*J*) was calculated as follows:

(2) $$\begin{array}{l} J=\text{PPFD}\times \Phi_{\text{PSII}}\times \alpha \times \beta \end{array}$$

where α and β are the leaf absorption and the proportion of quanta absorbed by PSII, respectively. The product α × β was determined from the slope of the relationship between Φ_PSII_ and the quantum efficiency of CO_2_ up take (Φ_CO2_), obtained by varying light intensity under non–photorespiratory conditions at <2% O_2_ (Valentini et al., 1995).

The variable *J* method was used to calculate *g*_m_ using the following equation:

(3) $$\begin{array}{l} g_{\text{m}}=\frac{A}{{\text{C}}_{i}-\frac{\Gamma^{*}\times \left(J\;+\;8\left(A\;+\;R_{\text{d}}\right)\right)}{J-4\left(A\;+\;R_{\text{d}}\right)}} \end{array}$$

where Γ^*^ and *R*_d_ are the CO_2_ compensation point in the absence of respiration and the mitochondrial respiration rate in the light, respectively. Γ^*^ and *R*_d_ were measured following the method of Laiskas described by Li et al. (2012). The data are presented in Table S1.

The chloroplastic CO_2_ concentration (C_c_) was calculated as follows:

(4) $$\begin{array}{l} {\text{C}}_{\text{c}}=C_{\text{i}}-\frac{A}{g_{\text{m}}} \end{array}$$

The total conductance of CO_2_ was calculated as follows:

(5) $$\begin{array}{l} g_{t}=\frac{{g_{\text{s},\text{CO}2}}^{*}g_{\text{m}}}{g_{\text{s},\text{CO}2}+g_{\text{m}}} \end{array}$$

where *g*_s,co2_ is stomatal conductance to CO_2_, which was calculated as follows:

(6) $$\begin{array}{l} g_{\text{s},\text{CO}2}=\frac{A}{{\text{C}}_{\text{a}}-{\text{C}}_{\text{i}}} \end{array}$$

#### Dynamic gas exchange measurements

The response time of the gas exchange apparatus was checked before the measurement of the dynamic gas exchange, and a quick response time of 5 s at a flow rate of 500 mL min^−1^ was observed, which was similar to that in other studies (Leakey et al., 2002, 2003). The photosynthetic induction process was measured according to the procedure described in Figure S1 and by Sun et al. (2016, 2017). Briefly, seedlings were kept in darkness by placing them in a controlled growth chamber (PPFD 0 μmol m^−2^ s^−1^; temperature 28°C; relative humidity 60%; CO_2_ concentration 400 μmol mol^−1^) from 20:00 on the previous day until the measurement was started at 09:00. After a prolonged low-light exposure of 100 μmol m^−2^ s^−1^ (>15 min), the data were automatically recorded for 3 min. Thereafter, the PPFD in the chamber was set to nine different 3-min flecks of 1,500 μmol m^−2^ s^−1^ separated by 1-min low-light periods of 100 μmol m^−2^ s^−1^. The maximum *A* during the procedure was referred to as *A*_max−fleck_, and the steady-state *A* at the low-light phase was referred as *A*_min−fleck_. Times to 50 and 90% of the maximum photosynthetic rate (*T*_50%*A*_ and *T*_90%*A*_, respectively) were identified as the period between the start of the first high-light level and the time when the first data point exceeded each of the values in turn. Times to 50 and 90% of the maximum *g*_s_ (*T*_50%*gs*_ and *T*_90%*gs*_) were calculated similarly.

The integrated carbon gain was calculated as the integrated photosynthesis within 36 min from shifting to a high light level to the end of the ninth low-light period. The potential carbon gain should be calculated as $\overset{\text{n}}{\underset{1}{\sum }}\left(3\times A_{\text{sat}}+A_{\text{initial}}\right)$, where n is the number of flecks in Figure S1, which is 9 in the present study. The carbon loss is the difference between the potential and integrated carbon gains.

### Measurements of leaf hydraulic conductance (*K*_leaf_) and leaf water potential (Ψ_leaf_)

One day before the measurement of *K*_leaf_, the seedlings were moved to a controlled growth chamber to avoid environmental fluctuation and midday depression of photosynthesis. The environmental conditions were controlled as above. The *K*_leaf_ was measured from 9:00 to 11:00 using the evaporative flux method (Sack and Scoffoni, 2012). Briefly, the newest fully expanded leaves were excised with a fresh razor blade and then immediately recut under water. Then, the leaf was connected to silicone tubing with a compression fitting under water to prevent air entering the system. The tubing connects the leaf to a hard tube connected to a graduated cylinder on a balance capable of reading 0.1 mg. The balance logs data every 30 s to a computer. The measurement was conducted in a temperature-controlled growth chamber, where the air temperatures were controlled at either 28 or 40°C with a PPFD of 500 μmol m^−2^ s^−1^. The temperature of water, that was absorbed by leaves, was also adjusted to either 28 or 40°C. After equilibration to a steady state, which requires ~30 min after excising the leaves, the transpiration rate was calculated and leaf temperature was recorded using a thermometer (AZ Instrument Corp. Ltd). The leaf temperatures were 40.70 ± 0.30 and 28.89 ± 0.43°C, respectively. Then, the leaf area was immediately measured using a leaf area meter (LI-Cor 3000C, LI-COR, NE, USA), which can be finished within 5 s. Afterwards, the Ψ_leaf_ of these leaves was measured using a WP4 Dewpoint Potentia Meter (Decagon, Pullman, WA, USA) without equilibration. The *K*_leaf_ was calculated as follows:

(7) $$\begin{array}{l} K_{\text{leaf}}=\frac{E}{0-{\text{Ψ}}_{\text{leaf}}} \end{array}$$

The measured Ψ_leaf_ above, which was shown in Figure S2, was only used for the calculation of *K*_leaf_, because the leaves for the measurements were rehydrated for ~30 min. The measured Ψ_leaf_ above showed a similar trend with the Ψ_leaf_, which was measured immediately after excising the leaves.

### Statistical analysis

Two-way analysis of variance (ANOVA) and the least-significant difference (LSD) multiple comparison test were used to assess each of the parameters using Statistix 9 software (Analytical Software, Tallahassee, Florida, USA).

## Results

### Effects of N supplies and temperatures on leaf photosynthesis

Leaf N and Rubisco contents of the newest fully expanded leaves are presented in Table S2, which shows that the HN treatment significantly increased these contents (*P* < 0.05). The high N supply significantly increased *A*_sat_ and *g*_m,sat_ under both leaf temperatures (*P* < 0.01), but significantly decreased C_i,sat_ at 28°C (*P* < 0.01; Table 1). The N supply had no significant effect on C_c,sat_. At 28°C, the *A*_sat_ and *g*_m,sat_ increased by 53.9 and 121.0%, respectively, under the HN treatment compared with the LN treatment.

**Table 1:** Effects of different N supplies, temperatures. and their interactions on steady-state photosynthesis (*A*_sat_), stomatal conductance (*g*_s,sat_), mesophyll conductance (*g*_m,sat_), intercellular CO_2_ concentration (C_i,sat_), and chloroplastic CO_2_ concentration (C_c,sat_).

**N**	**T (°C)**	***A*_sat_ (μmol m^−2^ s^−1^)**	***g*_s,sat_ (mol m^−2^ s^−1^)**	**C_i,sat_ (μmol mol^−1^)**	***g*_m,sat_ (mol m^−2^ s^−1^)**	**C_c,sat_ (μmol mol^−1^)**	***g*_t,sat_ (mol m^−2^ s^−1^)**
LN	28	20.4 ± 0.8c	0.414 ± 0.040a	298 ± 3a	0.167 ± 0.012bc	174 ± 10a	0.091 ± 0.002b
	40	14.5 ± 1.3d	0.263 ± 0.050b	277 ± 8b	0.133 ± 0.028c	163 ± 17a	0.062 ± 0.009c
HN	28	31.4 ± 1.7a	0.460 ± 0.080a	257 ± 14c	0.369 ± 0.026a	173 ± 8a	0.137 ± 0.009a
	40	26.4 ± 4.1b	0.520 ± 0.120a	279 ± 6b	0.241 ± 0.025b	182 ± 12a	0.105 ± 0.006b
**ANOVA**							
	T	^**^	^**^	ns	^***^	ns	^***^
	N	^***^	^**^	^**^	^***^	ns	^***^
	T*N	ns	ns	^**^	^*^	ns	ns

The data were presented as the means ± SD of three replications; the data followed by different letters are significant at the P < 0.05 level. ^*^, ^**^, and ^***^ represent significant at P < 0.05, P < 0.01, and P < 0.001, respectively, and ns represent not significant at the P < 0.05 level. T, temperature; N, nitrogen.

**Figure 1:**
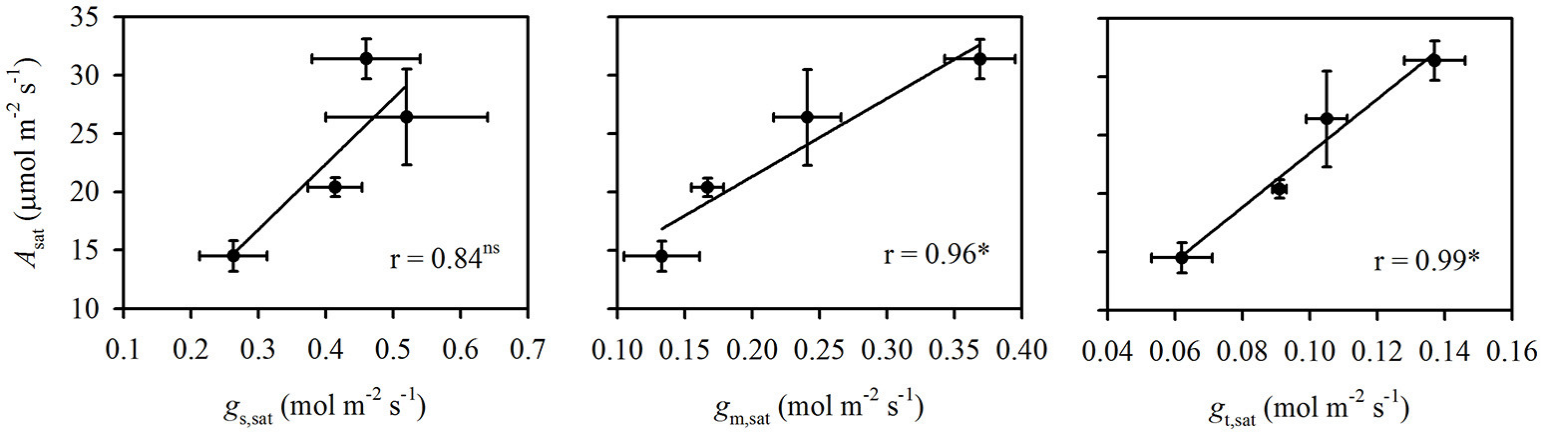
The correlations of *A*_sat_ vs. *g*_s,sat_, *g*_m,sat_, and *g*_t,sat_. The data are the means ± SD of three replications. *Represents significance at the *P* < 0.05 level, while ns represents no significance at the *P* < 0.05 level.

High temperature stress significantly decreased *A*_sat_ (*P* < 0.01) and *g*_m,sat_ (*P* < 0.001) under both N supplies. The decrease in *A*_sat_ under high temperature stress was lower in the HN treatment (15.9%) than in the LN treatment (28.9%). In contrast, the decrease in *g*_m,sat_ under high temperature stress was larger in the HN treatment (34.7%) than in the LN treatment (20.4%). The effects of temperature on *g*_s,sat_ and C_i,sat_ were dependent on N supply. High temperature stress significantly decreased *g*_s,sat_ (*P* < 0.05) in the LN treatment (36.5%), but it had no significant effect on that of the HN treatment. Additionally, the high temperature stress significantly increased C_i,sat_ in the HN treatment (*P* < 0.05), but significantly decreased it in the LN treatments (*P* < 0.05). The decrease in *g*_t,sat_ was higher under the LN treatment (31.9%) than under HN (23.4%). The N and temperature treatments had no significant effect on C_c,sat_. The *A*_sat_ was significantly and positively related to both *g*_m,sat_ and *g*_t,sat_ (*P* < 0.05), but was not related to *g*_s,sat_ (Figure 1).

### Effects of N supplies and temperatures on *K*_leaf_, Ψ_L_, and *E*

High N supply significantly increased *K*_leaf_ and *E* under both leaf temperatures (*P* < 0.001), although it was not significant for *E* between LN and HN at 28°C (Table 2). Ψ_Leaf_ was more negative in HN than in LN treatment at 28°C, in reverse, it was less negative in HN than in LN at 40°C.

**Table 2:** Effects of different N supplies and temperatures on leaf hydraulic conductance (*K*_leaf_), leaf water potential (Ψ_L_) and leaf transpiration rate (*E*).

**N**	**T (°C)**	***K*_leaf_ (mmol m^−2^ s^−1^ MPa^−1^)**	**Ψ_L_ (MPa^−1^)**	***E* (mmol m^−2^ s^−1^)**
LN	28	0.64 ± 0.21c	−0.72 ± 0.01a	5.81 ± 1.43c
	40	0.73 ± 0.12c	−1.48 ± 0.04c	10.03 ± 1.05b
HN	28	1.19 ± 0.18b	−1.07 ± 0.09b	7.17 ± 0.64c
	40	2.06 ± 0.32a	−1.01 ± 0.06b	16.08 ± 1.34a
**ANOVA**				
	T	^**^	^***^	^***^
	N	^***^	ns	^***^
	T*N	^*^	^***^	^**^

The data were presented as the means ± SD of three replications; the data followed by different letters are significant at the P < 0.05 level. ^*^, ^**^, and ^***^ represent significant at P < 0.05, P < 0.01, and P < 0.001, respectively, and ns represent not significant at the P < 0.05 level. T, temperature; N, nitrogen.

Two N supplies showed different responses of *K*_leaf_ and Ψ_Leaf_ to leaf temperature. High temperature stress significantly increased *K*_leaf_ in HN treatment (*P* < 0.01), but had no significant effect in LN treatment. In contrast, high temperature stress significantly decreased Ψ_Leaf_ in LN treatment (*P* < 0.05), but had no significant effect in HN treatment. High temperature stress significantly increased *E* under both N supplies (*P* < 0.001), it was increased by 73 and 124%, respectively, under LN and HN treatments.

### Effects of N supplies and temperatures on dynamic photosynthesis

**Figure 2:**
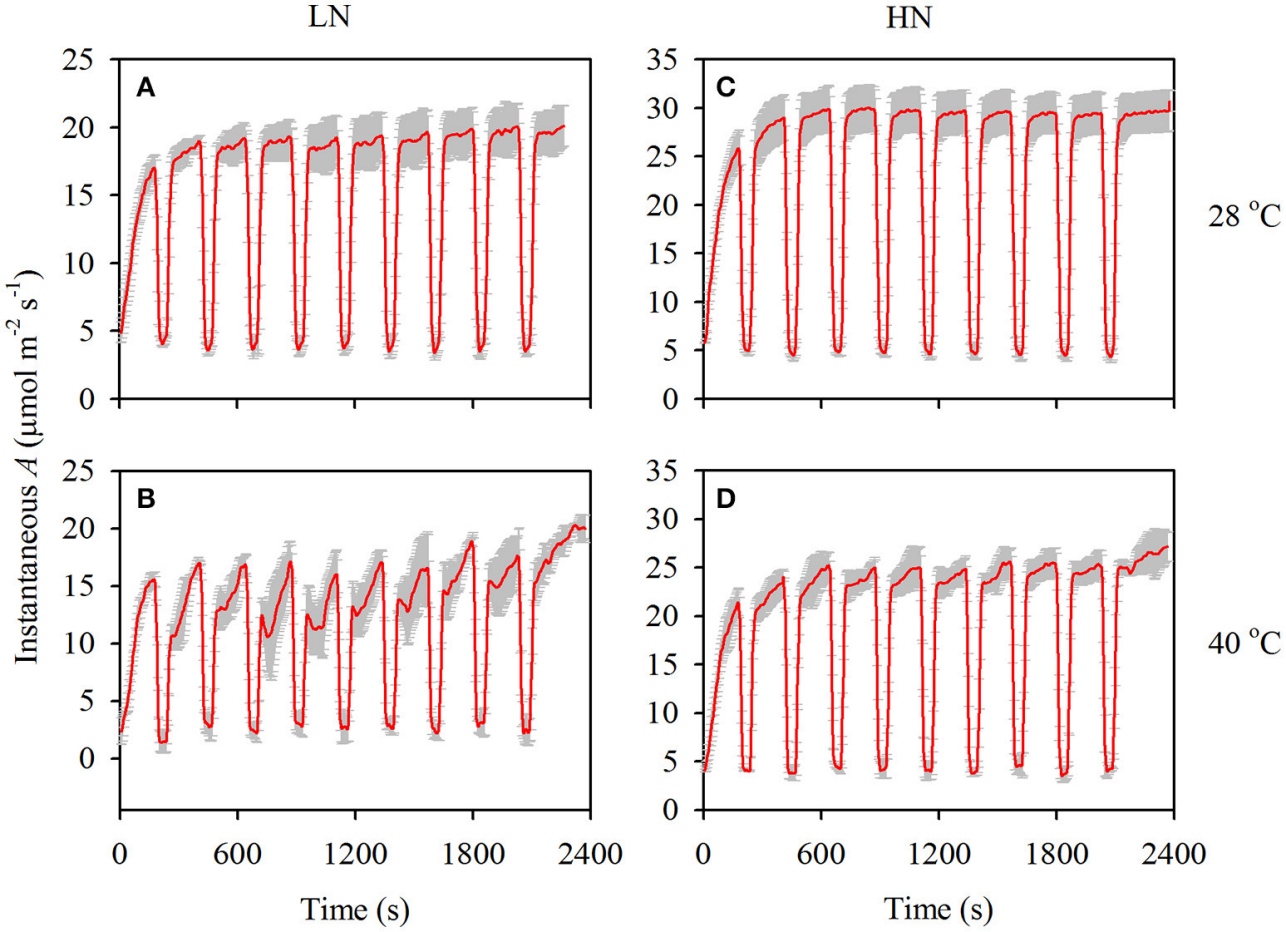
The responses of instantaneous photosynthesis **(A)** to flecked irradiance at 28 **(A** and **C)** and 40°C **(B** and **D)** under both low **(A** and **B)** and high **(C** and **D)** N supplies. The gray bars are SD of three replications.

After a prolonged (>15 min) low-light exposure of 100 μmol m^−2^ s^−1^, photosynthesis is gradually recovered when the leaves are shifted to a periodical high-light, and the induction process is shown in Figure 2. It can be observed that the increase of photosynthesis is faster in the HN than in the LN treatment, and high temperature stress significantly lowed the recovery rate. In fact, *T*_90%*A*_ was lower in the HN than in the LN treatment under both temperatures, and it was higher at 40°C than at 28°C under both N supplies (Table 3). The *T*_50%*A*_ value was also higher at 40°C than at 28°C under both N supplies, but the value was similar between the two N supplies. The different N supplies and temperatures had no significant effects on *T*_50%*gs*_, with the exception of a higher *T*_90%*gs*_ in the HN treatment at 40°C.

**Table 3:** Effects of different N supplies and temperatures on the times to 50 and 90% of maximum photosynthesis (*T*_50%*A*_ and *T*_90%*A*_, respectively) and of maximum stomatal conductance (*T*_50%*gs*_ and *T*_90%*gs*_, respectively) and on the maximum and minimum photosynthetic rates under flecks (*A*_max−fleck_ and *A*_min−fleck_, respectively).

**Parameters**	**LN**		**HN**		**T**	**N**	T*N
	**28°C**	**40°C**	**28°C**	**40°C**			
*T*_90%*A*_(s)	492.5 ± 195.2b	1850.2 ± 0.4a	302.0 ± 5.8c	612.3 ± 48.1b	^***^	^***^	^***^
*T*_50%*A*_ (s)	59.5 ± 13.1*b*c	78.2 ± 5.1a	44.3 ± 9.4c	73.3 ± 9.1*a*b	^**^	ns	ns
*T*_90%*gs*_(s)	256.0 ± 198.0b	118.2 ± 39.3b	247.2 ± 104.3b	1841.0 ± 13.4a	^***^	^***^	^***^
*T*_50%*gs*_(s)	66.3 ± 18.9a	62.7 ± 4.3a	56.2 ± 20.9a	68.0 ± 14.5a	ns	ns	ns
*A*_max−fleck_ (μmol m^−2^ s^−1^)	19.18 ± 1.09c	16.81 ± 1.16c	28.65 ± 2.37a	24.48 ± 1.32b	^**^	^***^	ns
*A*_min−fleck_ (μmol m^−2^ s^−1^)	4.03 ± 0.52a	2.47 ± 0.93b	4.39 ± 0.30a	4.09 ± 0.55a	^*^	^*^	ns
*A*_initial_ (μmol m^−2^ s^−1^)	4.99 ± 0.62ab	2.33 ± 1.09c	5.74 ± 0.09a	4.02 ± 0.17b	^***^	^*^	ns

The data were presented as the means ± SD of three replications; the data followed by different letters are significant at the P < 0.05 level. ^*^, ^**^, and ^***^ represent significant at P < 0.05, P < 0.01, and P < 0.001, respectively, and ns represent not significant at the P < 0.05 level. T, temperature; N, nitrogen.

The variation of *A*_max−fleck_ between different N and temperature treatments was similar to that of *A*_sat_, which was different from that of *A*_min−fleck_ (Tables 1, 3). High temperature stress significantly decreased the *A*_min−fleck_ and *A*_initial_ under the LN treatment (*P* < 0.05), but high temperature stress had no effect or less of an effect on them under the HN treatment. Moreover, the *A*_min−fleck_ and *A*_initial_ were similar between the LN and HN treatments at 28°C.

High N supply significantly increased the integrated and potential carbon gains (*P* < 0.001). However, high temperature decreased the integrated and potential carbon gains, although this was not statistically significant for the potential carbon gain (Table 4). The carbon loss was significantly higher under high temperature stress (*P* < 0.001), and was generally lower under the HN treatment.

**Table 4:** Effects of different N supplies and temperatures on integrated carbon gain, potential carbon gain, and carbon loss.

**N**	**T (°C)**	**Integrated carbon gain (μmol m^−2^)**	**Potential carbon gain (μmol m^−2^)**	**Carbon loss (%)**
LN	28	31,984 ± 1,808c	36,525 ± 1,386b	12.37 ± 5.49bc
	40	24,431 ± 2,331d	34,264 ± 972b	28.78 ± 5.16a
HN	28	47,465 ± 3,806a	51,041 ± 4,129a	7.01 ± 0.29c
	40	39,422 ± 2,381b	46,109 ± 2,923a	14.48 ± 1.81b
**ANOVA**				
	T	^***^	^*^	^***^
	N	^***^	^***^	^**^
	T^*^N	ns	ns	ns

The data were presented as the means ± SD of three replications; the data followed by different letters are significant at the P < 0.05 level. ^*^, ^**^, and ^***^ represent significant at P < 0.05, P < 0.01, and P < 0.001, respectively, and ns represent not significant at the P < 0.05 level.

## Discussion

### Steady-state photosynthesis

Under atmospheric CO_2_ concentration (380–400 ppm), C3 photosynthesis is mainly limited by Rubisco carboxylation and RuBP regeneration capacity (Farquhar et al., 1980; von Caemmerer and Evans, 2015). The former is determined by both CO_2_ diffusion capacity and Rubisco activity, while the latter is determined by the electron transport rate. It has been reported that N supply and temperature both can affect the rate-limiting steps for photosynthesis *via* changing the ratio of maximum electron transport rate to maximum Rubisco carboxylation capacity (*J*_max_/*V*_cmax_) and altering the N investment between Rubisco and electron transport proteins (Makino and Sage, 2007; Yamori et al., 2010, 2011). At optimal and supra-optimal temperatures, photosynthesis is limited by Rubisco under low N supply, while it is limited by RuBP regeneration under high N supply (Yamori et al., 2011). If photosynthesis is limited by Rubisco, such as under low N supply, the decrease in diffusion capacity will have a relatively larger constraint on photosynthesis; in contrast, the decrease in diffusion capacity will have a less negative effect on photosynthesis at the RuBP-regeneration step, such as under high N supply. This phenomenon is because the *A*/C_i_ response curve is steeper at the Rubisco-limited step and because Rubisco activity can be substantially affected by CO_2_ supply. This suggests that, with the same decrease in CO_2_ diffusion capacity, the decrease in photosynthesis would be more severe under low than under high N supply. In fact, the decrease in *g*_t,sat_ under high temperature stress was more severe under the LN (31.9%) than under the HN (23.4%) treatment (Table 1). Therefore, a larger decrease of *A*_sat_ under high temperature stress was observed in the LN treatment compared with the HN treatment. This point was also suggested by the close and positive correlation between *A*_sat_ and *g*_t,sat_ (Figure 1).

The larger decrease of *g*_t,sat_ under the LN treatment was mainly caused by the sharp decline in *g*_s,sat_ (Table 1). Stomatal conductance is closely related to leaf water status, especially Ψ_leaf_, which is the result of water balance between water loss through transpiration and water uptake. Water loss through transpiration would first increase with increasing leaf temperature, because the leaf-to-air water vapor pressure deficit increases and because the water viscosity decreases under high temperature (Mott and Peak, 2010). With an increased transpiration rate under high temperature, the capacity for water supply should substantially increase to avoid significant decreases in Ψ_leaf_ and *g*_t,sat_. Under the HN treatment, a substantially increased *K*_leaf_ value supports its increased transpiration rate and maintains its constant Ψ_leaf_ and *g*_s,sat_. In contrast, the *K*_leaf_ in the LN treatment did not significantly increase under high temperature, which resulted in significant decreases in Ψ_leaf_ and *g*_s,sat_ (*P* < 0.05, Table 2).

The reasons for the different responses of *K*_leaf_ to temperature under the two N supplies are not known and need to be further investigated. It has been reported that half of the apparent increase in hydraulic conductance can be explained by the decrease in water viscosity, and the remaining increase can be caused by the temperature dependence of outside-xylem hydraulic conductance, presumably through cell membranes (Matzner and Comstock, 2001; Sack et al., 2004; Mott and Peak, 2010). The permeability of cell membranes can be increased by increasing the amount and activity of aquaporins (Ishikawa-Sakurai et al., 2014; Ren et al., 2015). The activity of aquaporins and the permeability of the phospholipid bilayer are sensitive to temperature (Ahamed et al., 2012; Evans and von Caemmerer, 2013). Regarding this, the different responses of *K*_leaf_ to temperature under two N supplies are probably caused by the different contributions of outside-xylem components to hydraulic conductance, especially by the different water transport capacity through aquaporins. It should be noticed that *K*_leaf_ is sensitive to Ψ_leaf_, and *K*_leaf_ is usually lower in leaves with more negative leaf water potential (Guyot et al., 2012; Martorell et al., 2013). Under LN treatment at 40°C in the present study, the feedback effect of the decreased Ψ_leaf_, which was about 0.4 MPa more negative in comparison with HN treatment at 40°C (Table 2 and Figure S2), would probably be one of the reasons for its declined *K*_leaf_. Moreover, water potential measured with thermocouple psychrometer technique could potentially lead to more negative values compared to that with the pressure chamber (Barigah et al., 2014).

The temperature dependence of *g*_m_ differs among previous studies. Several studies have shown that *g*_m_ continuously increases with increasing temperature until 40°C (Scafaro et al., 2011; Evans and von Caemmerer, 2013). In contrast, many studies have suggested that *g*_m_ has an optimal temperature of ~30°C and that *g*_m_ significantly decreases below or above this threshold (Warren and Dreyer, 2006; Yamori et al., 2006; Warren, 2008; Walker et al., 2013). The temperature dependence of membrane permeability to CO_2_ is suggested to be the major reason for the change of *g*_m_ (Sage and Kubien, 2007; Evans and von Caemmerer, 2013). In the present study, high temperature stress resulted in significant decreases of *g*_m,sat_ under HN treatment both N supplies (*P* < 0.001, Table 1). Whether high N supply can lead to a more severe decrease in *g*_m,sat_ under high temperature stress should be verified.

### Dynamic photosynthesis

Photosynthesis will gradually increase after shifting from low to high light levels, and the *T*_50%*A*_ and *T*_90%*A*_ values are usually used to evaluate the rate of photosynthetic recovery. In the present study, *T*_50%*A*_ (*P* < 0.01) and *T*_90%*A*_ (*P* < 0.001) significantly increased under high temperature stress (Table 3). This increment suggests that high temperature stress significantly constrained the rate of photosynthetic recovery, especially under the LN treatment due to its larger increase in *T*_90%*A*_ (Table 3) and larger decrease in photosynthesis after each periodic low light condition (Figure 2).

The rate of photosynthetic recovery is mainly determined by the rates of both the reactivation of Rubisco and the reopening of stomata (Lawson et al., 2012). In the present study, the variation trends of *T*_90%*gs*_ and *T*_50%*gs*_ are different from those of *T*_90%*A*_ and *T*_50%*A*_ (Table 3). This suggests that the constrained recovery rate of photosynthesis under high temperature stress is not caused by the stomatal opening rate but rather by the delayed reactivation of Rubisco. In fact, Rubisco activase, which can facilitate carbamylation and maintenance of Rubisco activity, is thermolabile (Crafts-Brandner and Salvucci, 2000). After shifting to high light levels, the time required for the Rubisco activity and photosynthetic rate to reach a steady state is rapid in Rubisco activase-overexpressing plants, intermediate in wild-type plants, and slowest in antisense plants, especially under high temperature stress (Yamori, 2012).

Compared with steady-state conditions, CO_2_ assimilation will be significantly lower under flecked irradiances. It has been reported that daily carbon gain can be restricted by as much as 40% under flecked irradiance compared with uniform irradiance, and plant biomass can be severely lower under flecked irradiance (Leakey et al., 2002, 2005). Moreover, the inhibition of photosynthesis under flecked irradiance is more severe under drought, high temperature and nitrogen-deficient conditions (Leakey et al., 2003; Sun et al., 2016, 2017). In the present study, high temperature stress significantly aggravated (*P* < 0.001) the carbon loss under flecked light (Table 4). However, high nitrogen supply is available to offset the negative effect of high temperature on CO_2_ assimilation under flecked light.

## Conclusion

High N supply can significantly increase the tolerance of photosynthesis to high temperature stress *via* maintaining a constant Ψ_leaf_ and *g*_*s*_ by improving the *K*_leaf_. CO_2_ assimilation is significantly inhibited under flecked irradiance, and high temperature stress significantly aggravates this decrease. High N supply can significantly offset the constraint of high temperature on CO_2_ assimilation under flecked irradiance.

## Author contributions

YL conceived and designed the research. GH, QZ, and XW conducted the experiments and collected the data. YL and GH analyzed the data. YL and GH wrote the paper. SP commented and revised the paper. We would like to thank the editor and two reviewers for their useful comments to this paper.

### Conflict of interest statement

The authors declare that the research was conducted in the absence of any commercial or financial relationships that could be construed as a potential conflict of interest.
