# Supplementary material for: Nitrogen Can Alleviate the Inhibition of Photosynthesis Caused by High Temperature Stress under Both Steady-State and Flecked Irradiance
Source: Front Plant Sci. 2017 Jun 6;8:945. doi: 10.3389/fpls.2017.00945 (PMC5459896; doi:10.3389/fpls.2017.00945)
Supplement: Supplementary file 1 [file DataSheet1.DOCX]

Table S1 Effects of different N supplies and temperatures on the CO_2_ compensation point in the absence of respiration (*Γ**) and mitochondrial respiration rate in the light (*R*_d_).

| N | Temp.  (^o^C) | *Γ**  (μmol mol^-1^) | *R*_d_  (μmol m^-2^ s^-1^) |
| --- | --- | --- | --- |
| LN | 28 | 43.8±1.06b | 2.80±0.71a |
|  | 40 | 53.1±6.70a | 2.10±0.48ab |
| HN | 28 | 44.4±1.48b | 1.53±0.38b |
|  | 40 | 57.3±2.58a | 2.19±0.16ab |

Notes: Data were presented as means ± SD of three replications; Data followed by different letters are significant at *P* < 0.05 level.

Table S2 Effects of different N supplies on the leaf N and Rubisco content of the newest fully expanded leaves.

| Treatments | N  (g m^-2^) | Rubisco  (g m^-2^) |
| --- | --- | --- |
| LN | 0.974±0.055b | 3.092±0.448b |
| HN | 1.384±0.142a | 4.806±0.078a |

Notes: Data were presented as means ± SD of three replications; Data followed by different letters are significant at *P* < 0.05 level.

Fig. S1 The procedures of the photosynthetic induction process measurement under periodic high light.

Fig. S2 Effects of different N supplies and temperatures on the leaf water potential (Ψ_leaf_), which was measured after the measurement of leaf hydraulic conductance (*K*_leaf_).
